# Estrogen Receptor *β*1 Expression Patterns Have Different Effects on Epidermal Growth Factor Receptor Tyrosine Kinase Inhibitors’ Treatment Response in Epidermal Growth Factor Receptor Mutant Lung Adenocarcinoma

**DOI:** 10.3389/fonc.2020.603883

**Published:** 2021-01-29

**Authors:** Lijuan Zhang, Meng Tian, Jiamao Lin, Jianbo Zhang, Haiyong Wang, Zhenxiang Li

**Affiliations:** ^1^ Department of Pediatric Surgery, Shandong Provincial Hospital Affiliated to Shandong First Medical University, Jinan, China; ^2^ Department of Radiation Oncology, The First Affiliated Hospital of Nanjing Medical University, Nanjing, China; ^3^ Department of Medical Oncology, Shandong Cancer Hospital and Institute, Shandong First Medical University and Shandong Academy of Medical Sciences, Jinan, China; ^4^ Department of pathology, Shandong Cancer Hospital and Institute, Shandong First Medical University and Shandong Academy of Medical Sciences, Jinan, China; ^5^ Department of Radiation Oncology, Shandong Cancer Hospital and Institute, Shandong First Medical University and Shandong Academy of Medical Sciences, Jinan, China

**Keywords:** estrogen receptor *β*, lung adenocarcinoma, epidermal growth factor receptor (EGFR) tyrosine kinase inhibitors (TKIs), non-genomic mechanism, resistance

## Abstract

Estrogen receptor *β* (ER*β*) can regulate cellular signaling through non-genomic mechanisms, potentially promoting resistance to epidermal growth factor receptor (EGFR) tyrosine kinase inhibitors (TKIs). However, the mechanisms underlying the ER*β*-mediated resistance to EGFR TKIs remain poorly understood. In this study, we investigated the role of the interaction between ER*β*1 and ER*β*5 in non-genomic signaling in lung adenocarcinoma. We established PC9 cell lines stably overexpressing ER*β*1 or ER*β*1/ER*β*5. Immunofluorescence revealed that ER*β*5 overexpression partly retained ER*β*1 in the cytoplasm. Immunoblotting analyses revealed that EGFR pathway activation levels were higher in PC9/ER*β*1/5 cells than those in PC9/ER*β*1 or control PC9 cells. In the presence of estradiol, PI3K/AKT/mTOR pathway activation levels were higher in ER*β*1/5-expressing cells than those in ER*β*1-expressing cells. Additionally, PC9/ER*β*1/5 cells were less prone to the cytotoxic and pro-apoptotic effects of gefitinib compared with PC9/ER*β*1 or control PC9 cells. Cytoplasmic ER*β*1 was associated with poor progression-free survival in lung cancer patients treated with EGFR TKIs. These results suggest that cytoplasmic ER*β*1 was responsible for EGFR TKI resistance slightly through non-genomic mechanism in EGFR mutant lung adenocarcinoma.

## Introduction

Epidermal growth factor receptor (EGFR) tyrosine kinase inhibitors (TKIs) have revolutionized non-small cell lung cancer (NSCLC) personalized treatment and have improved the survival and quality of life of EGFR-mutant NSCLC patients (1–3). However, the development of primary and acquired resistance to TKIs remains a significant clinical challenge. Several mechanisms underlying acquired resistance to EGFR TKIs have been identified, including the acquisition of EGFR T790M mutation, c-MET amplification, PIK3CA mutations, and phenotypic transformation into small cell lung cancer (4, 5). Nevertheless, the mechanisms involved in primary resistance are poorly understood (5–8).

Estrogen receptor *β* (ER*β*) is the primary ER subtype expressed in lung cancer; upon binding to estrogen in the cytoplasm, ER*β* activates non-genomic signaling pathways, including PI3K/AKT/mTOR and RAS/RAF/MEK/MAPK pathways, promoting cancer cell proliferation and apoptosis evasion (9, 10). Importantly, significant overlap exists between ER*β*- and EGFR-regulated signaling pathways (11). Preclinical studies have shown that EGFR expression was downregulated in response to estradiol (E2); in contrast, ER*β* antagonists upregulated EGFR expression, highlighting the crosstalk between ER*β* and EGFR signaling (12). Hence, it is believed that non-genomic signaling events may modulate EGFR TKI resistance. ER belongs to the nuclear receptor superfamily of ligand-activated transcription factors. Since a nuclear localization of ER*β* in cancer cells has been reported (13), the relevance of cytoplasmic ER*β* in non-genomic signaling activation in cancer cells has attracted increasing attention in recent years.

Studies on endocrine-related cancers suggested that certain ER*β* isoforms are associated with ER*β* protein localization and patient prognosis (14–17). For example, ER*β*1 (also known as wild-type ER*β*) was primarily found in the nucleus of prostate cancer cells, whereas ER*β*5 localized both in the cytoplasm and nucleus (16, 18, 19). Although ER*β*5 lacks the ability to bind estrogen or form homodimers due to the absence of helix 12 in its C-terminal, it can heterodimerize with ER*β*1 in the presence of estrogen (20).

The aim of this study was to assess the role of the interaction between ER*β*1 and ER*β*5 in non-genomic signaling in lung adenocarcinoma. To this end, we overexpressed ER*β*1 and ER*β*5 in EGFR exon 19 deletion-harboring lung adenocarcinoma cells and assessed their ability to form heterodimers, as well as the relevance of ER*β*1/ER*β*5 heterodimerization in non-genomic signaling and response to EGFR TKIs.

## Materials and Methods

### Cell Culture and Chemicals

The EGFR-mutant lung adenocarcinoma cells PC9, HCC827, H1975, and H1650 were kindly provided by Peking University Cancer Hospital. Cells were cultured in RPMI-1640 medium supplemented with 10% fetal bovine serum and maintained at 37°C in a humidified 5% CO_2_ atmosphere.

Gefitinib was purchased from Selleck Chemicals (Selleck, USA) and diluted in dimethyl sulfoxide (DMSO) at a concentration of 10 mmol/L. Estradiol (E2) was purchased from Sigma-Aldrich (Sigma-Aldrich, Germany) and diluted in pure ethanol at a concentration of 10 mmol/L. Both drugs were aliquoted and stored at −80°C.

### RNA Extraction and Quantitative Real-Time PCR

Total RNA was extracted using the RNAsimple Total RNA kit (Tiangen, China), and first-strand cDNA synthesis was performed using the PrimeScript™ RT Master Mix (Takara, Japan). The relative mRNA levels of ER*β*1 and ER*β*5 were measured using SYBR green PCR assays (Thermo Fisher Scientific, USA). The sequences of the primers used in qRT-PCR were as follows: ER*β*1 forward primer: 5′-GTCAGGCATGCGAGTAACAA-3′, reverse primer: GGGAGCCCTCTTTGCTTTTA; ER*β*5 forward primer: 5′-TGGTCACAGCGACCCAGGATG-3′, reverse primer: 5′-TTAGGGCGCGTACCTCGCATG-3′; GAPDH forward primer: 5′-GACCCCTTCATTGACCTCAAC-3′, reverse primer: 5′-CTTCTCCATGGTGGTGAAGA-3′. Cycle threshold (Ct) values were determined using the system and analysis software. The relative mRNA levels were determined by normalizing to the GAPDH mRNA levels.

### Establishment of Cell Lines Stably Expressing Estrogen Receptor *β*1 and Estrogen Receptor *β*1/5

Lentiviral vectors expressing ER*β*1 and ER*β*5 were purchased from GenePharma (Shanghai, China). PC9 cells were infected with lentiviruses (MOI = 50) for three days. Subsequently, transduced cells were selected with 2 μg/ml of puromycin for one week. ER*β*1-overexpressing single-cell clones were established (hereafter referred to as PC9/ER*β*1 cells), and stable ER*β*1 overexpression was confirmed by Western blot and qRT-PCR. PC9/ER*β*1 cells were then infected with viruses carrying ER*β*5 open reading frame (ORF), followed by selection with neomycin (600 mg/ml) for one week.

### Immunofluorescence

ERβ expression was assessed by immunofluorescence (IF). Cells were fixed with 4% paraformaldehyde at room temperature for 20 min, followed by incubation with 0.5% Triton X-100 for 15 min. Non-specific binding was blocked with 5% bovine serum at 37°C for 30 min, before incubating with anti-ER*β* (1:100; GeneTex, Cat No.: GTX70174, USA) and anti-ER*β*1 (1:200; Abcam, Cat No.:ab187291, USA) primary antibodies. After incubation at 4°C overnight, cells were incubated with a secondary antibody conjugated with Alexa Fluor^®^ 488 (1:500, Cell Signaling Technology, USA) for 1 h at room temperature. Subsequently, cell nuclei were counterstained with 4′,6-diamidino-2-phenylindole (DAPI), and samples were imaged using a confocal laser scanning microscope (Zeiss, Germany).

### Immunoblotting Analysis

Cells were cultured in serum-free medium for 24 h and then treated with gefitinib (40 nM) or/and estradiol (20 nM) for another 8 h. Total protein was extracted from cells using cell lysis buffer (Beyotime, China) supplemented with a protease inhibitor cocktail (Roche, Germany); protein concentration was determined using the BCA protein assay (Beyotime, Beijing, China). The following primary antibodies (1:1,000) were used: anti-EGFR, anti-phospho-EGFR (Tyr1068), anti-AKT, anti-phospho-AKT (Ser473), anti-RPS6, anti-phosphor-RPS6 (Ser235/236), anti-P21, anti-CyclinD3, anti-cleaved-PARP (cPARP), and anti-beta-actin (Cell Signaling Technology, USA). Membranes were then incubated with peroxidase-linked anti-mouse or anti-rabbit secondary antibodies (1:5,000; Cell Signaling Technology, USA) for 2 h at room temperature. The expression levels of indicated proteins were quantified using quantity one.

### Cell Viability and Colony Formation Assays

Cells were treated with estradiol (20 nM) during the experiment. Cell viability was assessed using a cell counting kit-8 (CCK8; Dojindo, Japan). Briefly, cells were seeded (3 × 10^3^ cells/well) in sextuplicate in 96-well plates containing 100 μl medium and incubated for 24 h. Subsequently, cells were treated with increasing concentrations of the indicated drugs for an additional 72 h. After treatment, 10 μl of water-soluble tetrazolium salt (WST-8) was added to each well and incubated for 2 h. Optical absorbance at 450 nm was measured using a microplate reader. Relative viability was calculated using the following formula: Relative viability (%/control) = [A450 (treated) − A450 (blank)]/[A450 (control) − A450 (blank)].

For colony formation assays, cells were seeded into 6 cm cell culture dishes (500 cells/dish) and treated for two weeks with 40 nM gefitinib or DMSO (1/1,000 dilution). After washing twice with phosphate-buffered saline (PBS), cells were stained with crystal violet (Beyotime, China) for 20 min and washed with PBS.

### Patients

The data from 103 Chinese patients with advanced lung adenocarcinoma were retrospectively reviewed. The inclusion criteria used for patient enrollment were as follows: (1) Pathological diagnosis of adenocarcinoma; (2) sufficient tissue for both EGFR and KRAS mutation detection and ER*β*1 immunohistochemistry; (3) presence of EGFR mutations associated with sensitivity to EGFR TKIs, including 19 exon deletion and 21 exon point mutation, and absence of EGFR T790M or KRAS mutations; (4) patients treated with EGFR TKIs, including erlotinib, gefitinib, and icotinib; (5) available clinicopathological characteristics, including sex, age, disease stage, and smoking history. Treatment responses were classified according to the response evaluation criteria in solid tumors (RECIST), version 1.1. Progression-free survival (PFS) time was defined as the time between the first day of EGFR TKI treatment until radiologic progression or death. The study was approved by the Ethics Review Committee of the Shandong Cancer Hospital.

### Epidermal Growth Factor Receptor and KRAS Mutation Detection and Immunohistochemistry for Estrogen Receptor *β*1

Amplification refractory mutation system (ARMS) was employed to detect different genetic variants, including EGFR (exon 19 deletions, L858R, and T790M) and KRAS mutations.

ER*β*1 expression in lung adenocarcinoma tissue samples was assessed by immunohistochemistry (IHC). Informed consent to use biopsy tissues was obtained from all patients. Briefly, formalin-fixed, paraffin-embedded tissue sections (3 μm) were deparaffinized and stained according to standard procedures. Sections were probed with anti-ER*β*1 mouse antibody (1:200; Abcam, USA); a biotinylated anti-mouse IgG secondary antibody was used. Brown staining in over 10% cancer cells with cytoplasm or/and nucleus was considered positive. No staining was observed in negative controls, including lung tissues probed with a non-immune primary antibody. Based on the localization of “positive” immunoreactivity in the cytoplasm, nucleus, or both, patients were grouped as cER*β*1-, n/cER*β*1-, or nER*β*1-positive. IHC staining was evaluated independently by two investigators (LZ and MT) and a pathologist (JZ).

### Statistical Analysis

Differences in the relative mRNA levels, cell viability, and apoptosis between different cell lines were analyzed using two-tailed Student’s t-tests. Difference of ER*β*1 expression between male and female was analyzed using chi-square test. Patients’ survival was estimated using the Kaplan–Meier method, and comparisons between groups were conducted using log-rank tests. All statistical tests were two-tailed, and P-values <0.05 were considered statistically significant. All statistical analyses were performed using GraphPad Prism 8.0 (Prism Software Inc., San Diego, USA).

## Results

### Estrogen Receptor *β*5 Affects Estrogen Receptor *β*1 Localization in Epidermal Growth Factor Receptor-Mutant Lung Adenocarcinoma Cancer Cells

**Figure 1:**
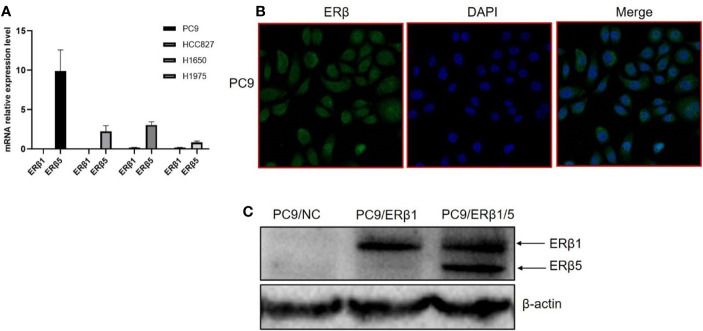
Construction of EGFR mutant lung cancer cell lines stably expressing ER*β*1 and ER*β*1/5. **(A)** mRNA level of ER*β*1 and ER*β*5 in four lung adenocarcinoma cell lines harboring EGFR mutation using specific primers determined by qRT PCR. Data shown as mean ± SD. **(B)** ER*β* expression pattern was assessed by immunofluorescence in PC9. **(C)** Western blot for detecting the expression level of ER*β* after stable transfection of ER*β*1 and ER*β*5. The upper stand refers to ER*β*1 while the lower is ER*β*5.

ER*β*5 has been identified as the predominant ER*β* splice variant in non-malignant lung tissues. In this study, we found that ER*β*5 mRNA levels were elevated in four lung adenocarcinoma cell lines harboring EGFR mutations (**Figure 1A**). We further assessed the role of ER*β*5 in lung adenocarcinoma using PC9 cells, which harbor EGFR exon 19 deletions. Immunofluorescence analyses revealed that endogenous ER*β* predominantly localized in the cell cytoplasm, and only low ER*β* levels were detected in the nucleus (**Figure 1B**).

**Figure 2:**
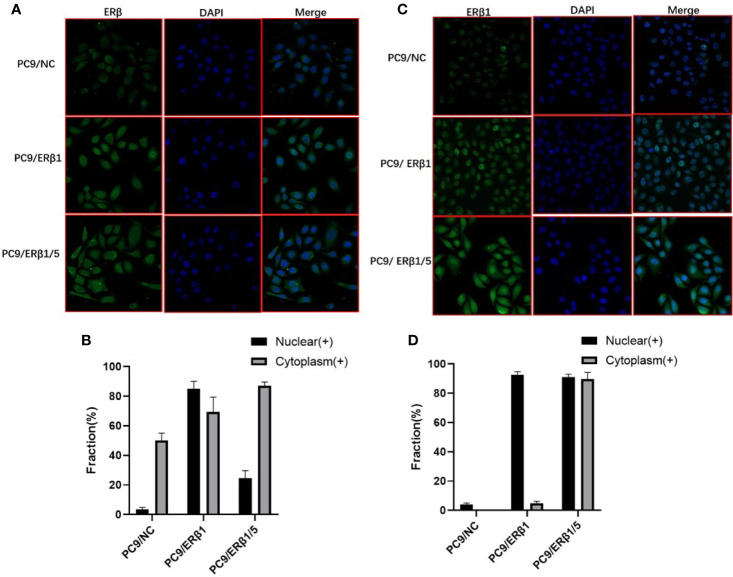
Different intracellular localization of ER*β* in three cell lines (PC9/NC, PC9/ER*β*1 and PC9/ER*β*1/5. **(A)** ERβ expression was analyzed in established cell lines using immunofluorescence by using primary antibodies against ER*β*. **(B)** The fraction of nuclear and cytoplasm ER*β* positive cells in three cell lines. **(C)** ER*β*1 expression was detected in the cell lines using immunofluorescence by using primary antibodies against ER*β*1. **(D)** The fraction of nuclear and cytoplasm ER*β*1 positive cells in three cell lines.

Next, we overexpressed ER*β*1 in PC9 cells (PC9/ER*β*1); we also overexpressed ER*β*5 in PC9/ER*β*1 cells (hereafter referred to as PC9/ER*β*1/5). ER*β*1 and ER*β*5 overexpression was confirmed at the mRNA and protein levels by qRT-PCR and immunoblotting, respectively (**Figure 1C** **;** **Table 1**). Immunofluorescence using a non-variant specific antibody revealed that ER*β* levels were elevated both in PC9/ER*β*1 and PC9/ER*β*1/5 cells; however, ER*β* localization differed between the two cell lines. Although ER*β* primarily localized in the cell nucleus in PC9/ER*β*1 cells, in PC9/ER*β*1/5 cells, it was found both in the cytoplasm and nucleus (**Figures 2A, B**).

**Table 1:** ΔCT value of ER*β*1 and ER*β*5 in indicated cell lines.

	ERβ1 ΔCT (mean ± SD)	P value	ERβ5 ΔCT (mean ± SD)	P value
PC9/NC	22.59 ± 1.33		13.33 ± 0.37	
PC9/ERβ1	2.73 ± 0.05	<0.01^*^	13.15 ± 0.27	
PC9/ERβ1/5	2.25 ± 0.03	<0.01^*^	5.84 ± 0.14	<0.01^#^

^*^The value was compared to PC9/NC. ^#^The value was compare to PC9/NC and PC9/ERβ1 respectively.

ER*β*1 has the highest affinity for estradiol among all ER*β* splice variants. Hence, we used an *ERβ1*-specific antibody to determine ER*β*1 localization. ER*β*1 predominantly localized in the cell nucleus in PC9/ER*β*1 cells. However, in PC9/ER*β*1/5 cells, we observed that ER*β*1 was partly detained in the cytoplasm, suggesting that the expression of ER*β*5 suppressed ER*β*1 translocation from the cytoplasm to the nucleus (**Figures 2C, D**).

### The Interaction Between Estrogen Receptor *β*1 and Estrogen Receptor *β*5 Regulates Downstream Signaling Events in the Presence of Estradiol

**Figure 3:**
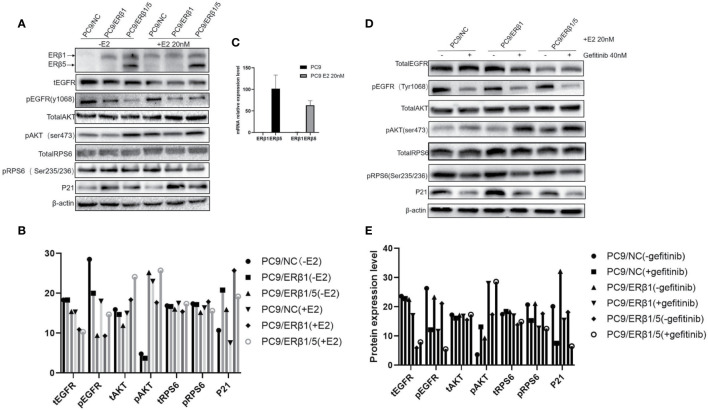
Interaction between ER*β*1 and ER*β*5 has a complex impact on downstream signaling under stimulation of estradiol. **(A)** Western blot was used to detect the indicated proteins involved in PI3K/AKT/mTOR signal pathway in the presence or absence of estradiol (20 nM). P21 was selected as marker for detecting ER*β* nuclear transcriptional activity. **(B)** Indicated protein expression using quantity one for **(A)**. **(C)** Relative mRNA expression level of ER*β*1 and ER*β*5 in the presence or absence of estradiol for PC9/NC. **(D)** The indicated cell lines were treated with gefitinib (40 nM) for 8 h in the presence of estradiol. Cell extracts were immunoblotted to detect the indicated proteins. **(E)** Indicated protein expression using quantity one for **(D)**.

Next, we assessed the role of nuclear and cytoplasmic ER*β* in transcriptional regulation and non-genomic signaling, respectively. The expression of the cell cycle regulator P21 is induced by the nuclear ER*β* (18). In this study, we found that P21 expression levels were profoundly higher in PC9/ER*β*1 cells compared to those in PC9/NC or PC9/ER*β*1/5 cells, especially after stimulation with estradiol (**Figure 3A**).

PI3K/AKT/mTOR signaling pathway is regulated by both EGFR and ER*β* (11). To determine the PI3K/AKT/mTOR pathway activation status, we assessed both total and phosphorylated levels of EGFR, AKT, and RPS6. We found that phospho-EGFR levels were lower in PC9/ER*β*1/5 cells than those in PC9/NC or PC9/ER*β*1 cells. Although total and phospho-EGFR levels decreased in all groups after estradiol treatment, the decrease in phospho-EGFR levels was stronger in PC9/ER*β*1 and PC9/ER*β*1/5 cells than that in PC9/NC cells. The phospho-AKT levels were higher in PC9/ER*β*1/5 cells than those in PC9/NC or PC9/ER*β*1 cells, both at baseline and after estradiol treatment. The levels of phospho-RPS6, which functions downstream of mTOR, were similar among the groups (**Figures 3A, B**).

We also found that ER*β*1 but not ER*β*5 was upregulated in PC9/NC cells after estradiol treatment. Interestingly, qRT-PCR showed no changes in the ER*β*1 mRNA levels after estradiol treatment, suggesting that the estradiol-mediated ER*β*1 upregulation occurs at the post-transcriptional level (**Figure 3C**).

When cells were treated with gefitinib in addition to estradiol, phospho-EGFR levels were decreased in all groups, whereas phosphor-AKT levels were increased, especially in PC9/ER*β*1 and PC9/ER*β*1/5 cells. Similar to phospho-EGFR, phospho-RPS6 levels were decreased in all groups after gefitinib treatment. P21 was also downregulated in gefitinib-treated cells (**Figures 3D, E**).

### PC9/Estrogen Receptor *β*1/5 Cells Are Less Prone to the Cytotoxic Effects of Gefitinib

**Figure 4:**
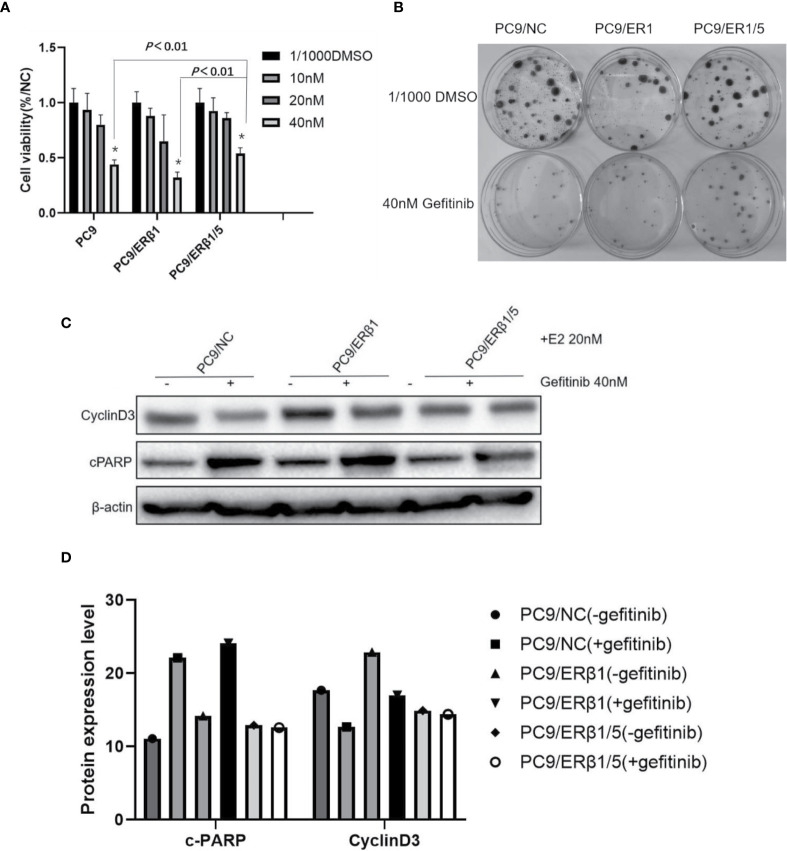
PC9/ER*β*1/5 showed less effective to gefitinib compared to that of PC9/NC and PC/ER*β*1 through anti-apoptosis effect. **(A)** Cell viability test for 72 h treatment at indicated concentration of Gefitinib (0, 10, 20, and 40 nM) in the presence of estradiol (20 nM). PC9/ER*β*1/5 cells were less effective to gefitinib compared to PC9/NC and PC9/ER1 cells at concentration 40 nM (^*^ *P* < 0.01). **(B)** Colony formation assay for 2 weeks in the presence of gefitinib 40 nM or 1/1,000 DMSO for three cell lines. **(C)** Cell apoptosis percentage after treatment of gefitinib (40 nM) for 24 h in the presence of estradiol (20 nM). Data shown as mean ± SD (^*^ *P* < 0.01). **(C)** Western blot for detecting cleaved PARP and CyclinD3 for three cell lines with or without gefitinib (40 nM) treatment under the stimulation of estradiol (20 nM). **(D)** Indicated protein expression using quantity one for **(C)**.

To determine the effects of different ER*β* splice variants in response to gefitinib, we performed cell viability and colony formation assays. We found that PC9/ER*β*1/5 cells were less prone to the cytotoxic effects of gefitinib (40 nM) compared with PC9/NC or PC9/ER*β*1 cells, although we found no significant differences in cell viability at low concentrations of gefitinib (**Figure 4A**). Additionally, the ability of gefitinib (40 nM) to inhibit colony formation was stronger in PC9/ER*β*1 and PC9/NC than in PC9/ER*β*1/5 cells (**Figure 4B**).

We found that cPARP levels were increased in all three groups after gefitinib treatment. However, the increase in cPARP levels was more substantial in PC9/NC and PC9/ER*β*1 cells than in PC9/ER*β*1/5 cells. Consistently, the decrease in cyclin D3 levels was more profound in PC9/NC and PC9/ER*β*1 cells, while almost no change in cyclin D3 levels was observed in PC9/ER*β*1/5 cells after gefitinib treatment (**Figures 4C, D**). These results suggest that PC9/ER*β*1/5 cells are less sensitive to EGFR TKIs than PC9/NC and PC9/ER*β*1 cells.

### Estrogen Receptor *β*1 Expression and Intracellular Distribution Affect Progression-Free Survival in Patients With Advanced Epidermal Growth Factor Receptor-Mutant Lung Adenocarcinoma

In this study, we retrospectively analyzed the data from 103 stage IIIb–IV lung adenocarcinoma patients treated with EGFR TKIs at the Shandong Cancer Hospital between January 2014 and November 2017. All patients harbored EGFR mutations affecting response to EGFR TKIs, including exon 19 deletions (47; 45.6%) and exon 21 point mutations (55; 53.4%); one patient had G719X mutations, whereas no EGFR T790M or KRAS mutations were detected. The clinicopathological characteristics of the patients are summarized in **Table 2**. Most patients were never/light smokers (79; 76.7%) and women (65; 63.1%).

**Table 2:** Clinical and pathological characteristics of 103 patients with EGFR mutant lung adenocarcinomas.

Variables	Number of cases (%)
Age, Years	
Median	55
Range	33–77
Gender	
Male	38(36.9)
Female	65(63.1)
Smoking status	
Ever or current	24(23.3)
Never	79(76.7)
Stage	
III	6(5.8)
IV	97(94.2)
EGFR mutation type	
19del	47(45.6)
L858R	55(53.4)
Other	1(1)
Response evaluation	
PR	56(54.4)
SD	43(41.8)
PD	2(1.9)
No Evaluation	2(1.9)
ERβ1 expression pattern	
Nuclear	39(37.9)
Cytoplasmic + Nuclear	24(23.3)
Cytoplasmic only	20(19.4)
Negative	20(19.4)

**Figure 5:**
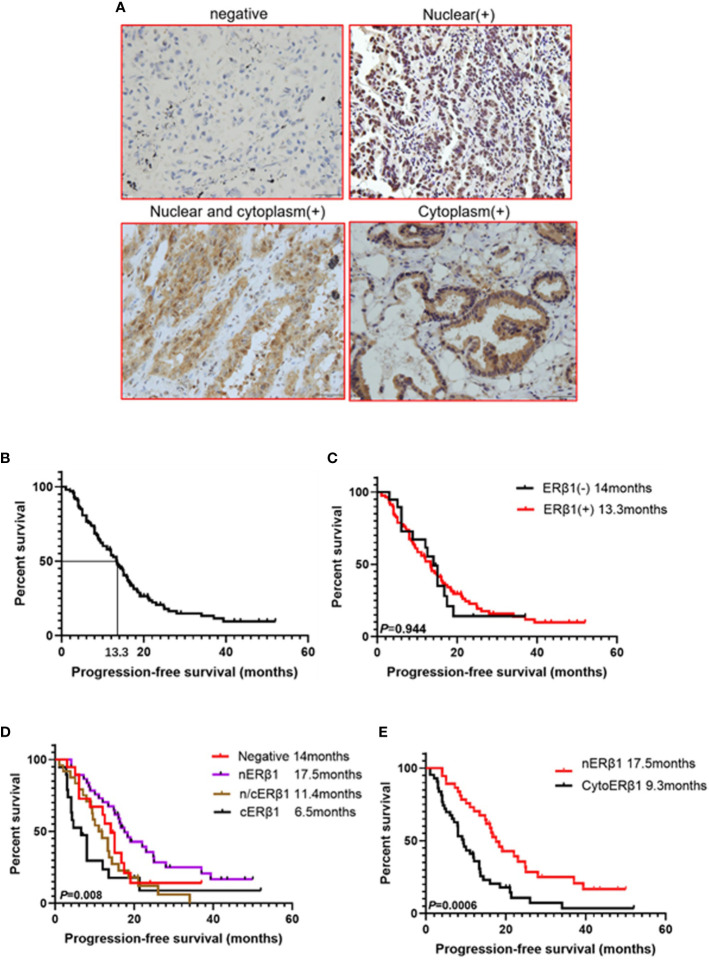
ER*β*1 expression pattern had different effect on progression-free survival in advanced EGFR mutant lung adenocarcinomas. **(A)** Representative ER*β*1 expression within lung adenocarcinoma tissues (Negative, nuclear, nuclear and cytoplasm, cytoplasm). **(B)**. Kaplan–Meier curve showed progression free survival for all the patients (median survival: 13.3 months). **(C)** Kaplan–Meier curves illustrated progression-free survivals in the groups with ER*β*1 positive (median survival:13.3 months) and ER*β*1 negative (median survival: 14 months) (log-rank test, *P* = 0.944). **(D)** Comparison of progression free survival in four groups using log-rank test (*P* = 0.008). **(E)** Comparison of progression free survival between nuclear ER*β*1 positive group (median survival: 17.5 months) and cytoER*β*1 positive group (median survival: 9.3 months) (log-rank test, *P* = 0.0006).

ER*β*1 expression was detected in 80.6% (83/103) of the patients, and the intracellular distribution pattern differed significantly (**Figure 5A**); strong nuclear accumulation (nER*β*1) was observed in 37.9% (39/103) of the patients, nuclear/cytoplasmic accumulation (n/cER*β*1) in 23.3% (24/103), and strong cytoplasmic accumulation (cER*β*1) in 19.4% (20/103) of the patients (**Table 2**). There was no difference of ER*β*1 pattern between male and female patients (**Table 3**).

**Table 3:** Expression pattern of ER*β*1 stratified by sex.

Expression pattern	Male (No.)	Female (No.)	X^2^	P value
Nuclear (+)	14	25		
Cytoplasmic+Nuclear(+)	5	19		
Cytoplasmic (+)	11	9		
Negative	8	12	5.575	0.13

At the time of data collection (Nov 20, 2017), four patients were lost to follow-up, and 79 patients (76.7%) presented with progressive disease. The median survival of the 99 patients was 13.3 months (**Figure 5B**). There was no significant difference in PFS between patients with ER*β*1-positive and ER*β*1-negative tumors (13.3 months *vs.* 14 months, *P* = 0.944) (**Figure 5C**). Interestingly, we also found significant differences in the median PFS of patients with different intracellular ER*β*1distribution pattern (nER*β*1, 17.5 months; n/cER*β*1, 11.4 months; cER*β*1, 6.5 months; negative, 14 months, *P* = 0.008) (**Figure 5D**). Because cytoplasmic ER*β*1 is key for non-genomic signaling activation, we combined patients with n/cER*β*1 expression and those with cER*β*1 (cytoER*β*1). Survival analysis showed that patients with cytoER*β*1 expression (n = 43) had a shorter median PFS after EGFR TKI treatment (9.5 months) compared to those with nER*β*1 expression (n =37; PFS, 17.5months; *P* = 0.0006) (**Figure 5E**).

## Discussion

Approximately 20–30% of patients with EGFR activating mutations exhibit primary resistance to EGFR TKIs. The mechanism underlying resistance to EGFR TKIs, and primary resistance, in particular, are extremely complex and remain poorly understood. ER*β* expression has been associated with response to EGFR TKIs. Notably, in a Japanese cohort study, strong ER*β* expression predicted favorable clinical outcomes in patients with lung adenocarcinoma after treatment with EGFR TKIs. In contrast, we previously identified high cytoplasmic ER*β* expression as a predictor of poor PFS (21, 22). Therefore, further elucidation of the expression pattern and intracellular distribution of ER*β* is required to determine the effects of non-genomic signaling on EGFR signal transduction and clinical outcomes.

Several ER*β* splicing variants have been identified, the most important of which are ER*β*1 (wild-type ER*β*), and ER*β*2–5 (20, 23). ER*β*1 is the only fully functional receptor in the ER*β* family, and has the highest affinity for estradiol; other ER*β* family members have weak to no ligand binding capacity, despite maintaining their ability to heterodimerize with ER*β*1 (20). Therefore, assessing the function of ER*β* splice variants other than ER*β*1 is equally important. Notably, the crucial role of ER*β*5 in lung cancer is becoming increasingly evident (17, 24).

In this study, we focused on the role of ER*β*1 and ER*β*5 in lung adenocarcinoma. Previous studies demonstrated that ER*β*1 was predominantly localized in the cell nucleus and exerted anti-proliferative effects. In contrast, ER*β*5 was found both in the cytoplasm and nucleus, and it has been implicated in cancer cell migration and invasion (17, 22, 25). Our results confirmed the elevated ER*β*5 levels in EGFR-mutant lung cancer cells; in contrast, ER*β*1 was lowly expressed. These results were consistent with those of a previous study showing that ER*β*5 was the primary ER*β* isoform expressed in non-malignant lung cells, and heterodimerized with ER*β*1 (20). Similarly, we previously showed that ER*β*5 formed complexes with ER*β*1, confirming their ability to interact (22).

In this study, we also found that ER*β*1 was predominantly localized in the cell nucleus. However, the forced overexpression of ER*β*5 partly retained ER*β*1 in the cytoplasm. Hence, the presence of ER*β*5 can explain previous findings of ER*β*1 localization in the nucleus and cytoplasm in cancer cells.

Total and phospho-EGFR levels were decreased after estradiol treatment, highlighting the crosstalk between EGFR and ER*β* signaling pathways (12). P21 is an essential cell cycle regulator, playing important tumor-suppressing roles (26). Importantly, P21 expression was induced by ER*β (* 18, 25). In this study, we confirmed that ER*β*1 increased P21 levels, suggesting a role of ER*β*1 in transcriptional regulation in lung cancer cells. Consistently, ER*β*1 exerted anti-proliferative effects in other cancer cells (15, 18). However, when ER*β*1 and ER*β*5 were co-expressed, P21 levels were lower compared with those in PC9/ER*β*1 cells, suggesting that ER*β*5 impairs the transcriptional abilities of ER*β*1. However, in the presence of estradiol, PI3K/AKT/mTOR signaling pathway activation levels were higher in ER*β*1/5-expressing lung cancer cells than those in ER*β*1-expressing cells, suggesting that the interaction between ER*β*1 and ER*β*5 potentiated the effects of ER*β*1 in non-genomic signaling. Hence, we believe that ER*β*1 translocation from the nucleus to cytoplasm in the presence of ER*β*5 was essential in determining its biological function, reflecting the bi-faceted role of ER*β* in cancer (27).

mTOR signaling was inhibited by gefitinib treatment in all groups, although phospho-AKT levels were increased. Consistently, gefitinib treatment exerted cytotoxic effects in all cell groups. However, ER*β*1/ER*β*5 co-expression rendered cells less prone to the cytotoxic and pro-apoptotic effects of gefitinib. These results confirmed the critical role of ER*β*1/ER*β*5 complexes in estrogen receptor-mediated non-genomic signaling. We also found that estradiol upregulated ER*β*1 but not ER*β*5 at the post-transcriptional level, confirming the high affinity of ER*β*1 for estradiol.

In this study, we also investigated the effect of the ER*β*1 expression pattern on PFS in EGFR-mutant lung adenocarcinoma patients. We found that patients with nuclear ER*β*1 expression exhibited a relatively longer PFS after EGFR TKI treatment, whereas cytoplasmic ER*β*1 was associated with shorter PFS after EGFR TKI treatment. These results highlight the clinical relevance of our findings from *in vitro* experiments in EGFR-mutant lung cancer cell lines. Importantly, nuclear ER*β*1 expression in lung cancer tissues was associated with tumor-suppressing effects, whereas cytoplasmic ER*β*1 promoted EGFR TKI resistance to some extent. Although cytoER*β*1 was associated with a lower response to EGFR TKIs, the median PFS of patients with cytoER*β*1 was 9.5 months, suggesting that EGFR mutations remain the most powerful predictor for EGFR TKI treatment response. The findings reported here need to be confirmed in large cohort prospective studies. Additionally, the relationship between ER*β*1/ER*β*5 ratio and response to EGFR TKIs merits further investigation. Our study has potential clinical relevance for guiding anti-estrogen agents’ treatment in future. Recently two clinical trials evaluating efficacy and safety of EGFR TKI plus anti-estrogen treatment showed no survival improvement in combination group compared to that of EGFR TKI alone (28, 29). However, the status of ER*β* expression and its predictive value for anti-estrogen therapy were not evaluated in both trials.

In conclusion, we showed that ER*β*1 localized in the cell cytoplasm by interacting with ER*β*5, inducing non-genomic signaling activation, and promoting EGFR TKI treatment resistance in EGFR-mutant lung adenocarcinoma. Hence, these results suggest that cytoplasmic ER*β*1 was responsible for EGFR TKI resistance slightly through non-genomic mechanism in EGFR mutant lung adenocarcinoma.

## Data Availability Statement

The original contributions presented in the study are included in the article/supplementary materials; further inquiries can be directed to the corresponding authors.

## Ethics Statement

The studies involving human participants were reviewed and approved by the Ethics Committee of the Shandong Cancer Hospital and Institute. The patients/participants provided their written informed consent to participate in this study.

## Author Contributions

LZ and MT did the experimental work and participated in the analysis of the results. ZL supervised the research and secured funding for the project. JZ interpreted the histological findings. HW and JL wrote the paper draft, and all authors contributed to the analysis of the results. All authors contributed to the article and approved the submitted version.

## Funding

This study was supported jointly by the National Natural Science Foundation of China (Grant No. 81602031), The Natural Science Foundation of Shandong Province (Grant No. ZR2016HB12), The National Natural Science Foundation of China (Grant No. 81904186), Special Funds for Taishan Scholars Project (Grant No. tsqn201812149), Academic Promotion Programme of Shandong First Medical University (Grant No. 2019RC004) and Key research and development plan project of Shandong Province (Grant No. 2017GSF18158).

## Conflict of Interest

The authors declare that the research was conducted in the absence of any commercial or financial relationships that could be construed as a potential conflict of interest.
